# Supplementary material for: Co-delivered PD-L1 rescues the protective efficacy mediated by an AAV-expressed HIV-1 bNAb
Source: bioRxiv. 2026 Jun 1:2026.05.29.728706. Preprint. [Version 1] doi: 10.64898/2026.05.29.728706 (PMC13252170; doi:10.64898/2026.05.29.728706)
Supplement: Supplement 1 [file NIHPP2026.05.29.728706v1-supplement-1.pdf]

bioRxiv preprint doi: https://doi.org/10.64898/2026.05.29.728706; this version posted June 1, 2026. The copyright holder for this preprint (which was not certified by peer review) is the author/funder, who has granted bioRxiv a license to display the preprint in perpetuity. It is made available under a CC-BY 4.0 International license.

# Supplementary Materials for

## Co-delivered PD-L1 rescues the protective efficacy mediated by an AAV-expressed HIV-1 bNAb

Michael Kuipa, Abubakarr A. Koroma, Isai Leguizamo, Priya Dhole, Yash Barot, Michelle Y.-H. Lee, Gregory K. Tharp, Shan Liang, Magdalen Chouinard, Stephanie Ehnert, Stacey Weissman, Casey Whitehead, Rachelle L. Stammen, Jennifer S. Wood, Elizabeth H. Curran, Deepa Machiah, Evan D. Dessasau, Yoshiaki Nishimura, Jun Xie, Guangping Gao, Sumit Verma, Deanna A. Kulpa, Ian N. Moore, Steven E. Bosinger, Matthew R. Gardner

Corresponding author: Matthew R. Gardner, matthew.r.gardner@emory.edu

**The PDF file includes:**

Figs. S1 to S6
Table S1

bioRxiv preprint doi: https://doi.org/10.64898/2026.05.29.728706; this version posted June 1, 2026. The copyright holder for this preprint (which was not certified by peer review) is the author/funder, who has granted bioRxiv a license to display the preprint in perpetuity. It is made available under a CC-BY 4.0 International license.

## Supplemental Figure Legends

**Fig. S1. AAV9 vectors encoding 3BNC117 or rhesus PD-L1.**
**(A)** Diagram of the AAV transgene cassette encoding 3BNC117. 3BNC117 was "rhesusized" by exchanging its native human constant regions for rhesus macaque constant regions. **(B)** Diagram of the AAV transgene cassette encoding PD-L1 (*Macaca mulatta*). Abbreviations: ITR, AAV2 inverted terminal repeat; CMVp, cytomegalovirus immediate-early promoter; CBAp, chicken-beta actin promoter; SV40 intron, simian virus 40 intron; $V_H$, variable heavy chain region; $rhC_H$. rhesus macaque (rh) constant heavy chain (IgG1); furin cleavage site, RKRR; SGSG linker, serine/glycine linker; P2A, ribosomal skipping peptide from porcine teschovirus-1; $V_L$, variable light chain region; $rhC_L$, rhesus macaque (rh) kappa light chain constant region; WPRE, woodchuck hepatitis virus posttranscriptional regulatory element; pA, SV40 polyadenylation signal sequence; kb, kilobases. Note the M428L/N434S half-life-extending amino acid substitutions in the $rhC_H$ region. The size of the AAV transgene cassettes is indicated on the right.

**Fig. S2. Macaque weight gain over the course of the study.**
Weight gain over the course of 52 weeks in macaques that received **(A)** AAV9.3BNC117 only, **(B)** AAV9.3BNC117 plus AAV9.PD-L1 or **(C)** AAV9.PD-L1 only.

**Fig. S3. Serum bNAb concentration vs ADA response.**
Serum bNAb concentrations vs ADA endpoint titers in individual macaques that received **(A)** AAV9.3BNC117 only or **(B)** AAV9.3BNC117 plus AAV9.PD-L1, measured by gp120 ELISA or RSC3 over 52 weeks. For ADA endpoint titers, serum collected 2 weeks prior to study initiation was used as baseline for each animal. ADA endpoint titers are defined as the highest serum dilution with $OD_{450} \geq 0.2$.

**Fig. S4. AAV9 neutralizing antibody responses and PBMC IFNγ ELISpot reactivity in macaques.**
AAV9 neutralization titers of all 18 macaques from Weeks -2, 0, 4, 10 and 50 post AAV9 administration. $ID_{50}$ values for each macaque per group are reported. Those samples that did not reach 50% neutralization were normalized to a value of <10. Serum collected 2 weeks prior to study initiation was used as baseline for each macaque. Serum $ID_{50}$ titers in individual macaques that received **(A)** AAV9.3BNC117 only, **(B)** AAV9.3BNC117 plus AAV9.PD-L1 or **(C)** AAV9.PD-L1 only. **(D)** $ID_{50}$ AUC values for data in (A–C). Black lines indicate the median. Note symbols in (D) match those used in panels (A–C), respectively. **(E)** IFNγ ELISpot reactivity for Weeks 0, 4, 20 and 52 PBMC samples against AAV9.3BNC117 peptide pools (3BNC117 VarH, 3BNC117 VarL, LS-Furin-P2A peptide, IgG1 constant heavy chain region, and kappa light chain constant regions) in macaques that received AAV9.3BNC117. **(F)** Comparison of PBMC IFNγ ELISpot reactivity against AAV9.3BNC117 peptide pools at week 52 in macaques that received AAV9.3BNC117 only or AAV9.3BNC117 and AAV9.PD-L1. Black lines indicate the median. Note symbols in (E) and (F) match those used in panels (A) and (B), respectively. Statistical significance in (F) was determined by two-tailed Mann-Whitney test and defined as $p \leq 0.05$.

bioRxiv preprint doi: https://doi.org/10.64898/2026.05.29.728706; this version posted June 1, 2026. The copyright holder for this preprint (which was not certified by peer review) is the author/funder, who has granted bioRxiv a license to display the preprint in perpetuity. It is made available under a CC-BY 4.0 International license.

**Fig. S5. Histopathology staining for macaques that received AAV9 administered vectors.** Representative H&E-stained muscle tissue from the upper left quadriceps of all 18 macaques that was harvested at necropsy. Stained tissue from individual macaques that received **(A)** AAV9.PD-L1 only, **(B)** AAV9.3BNC117 only, or **(C)** AAV9.3BNC117 plus AAV9.PD-L1. Regions of interest are enlarged on the right. Scale bars represent 1 mm on the left and 100 µm on the right.

**Fig. S6. TLSs develop in macaques with severe inflammation at the site of AAV administration.**
IHC staining for CD19 in **(A)** Mm001 and **(B)** Mm003 showing TLSs. Scale bars represent 100 µm.

**Table S1. Characteristics of the 18 rhesus macaques enrolled in this study.**

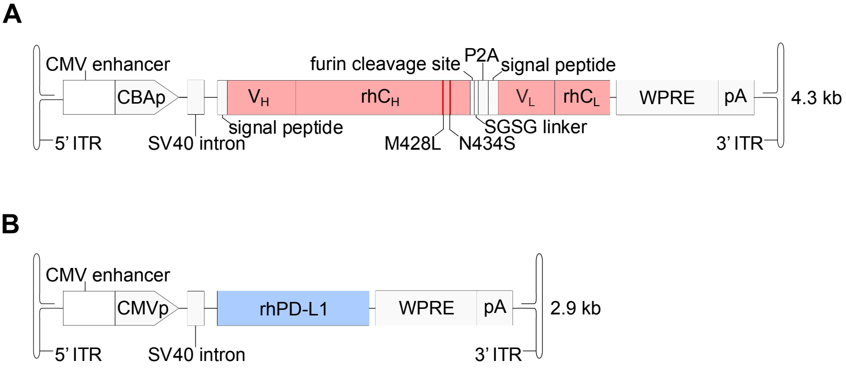


Kuipa et al., Fig. S1

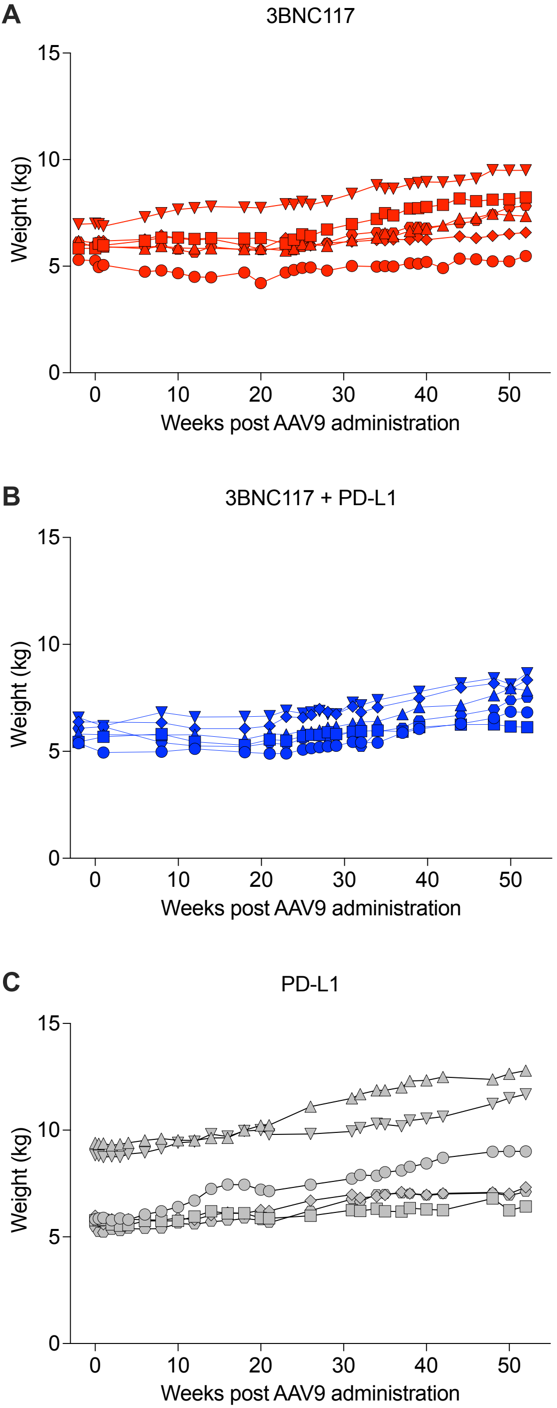


Kuipa et al., Fig. S2

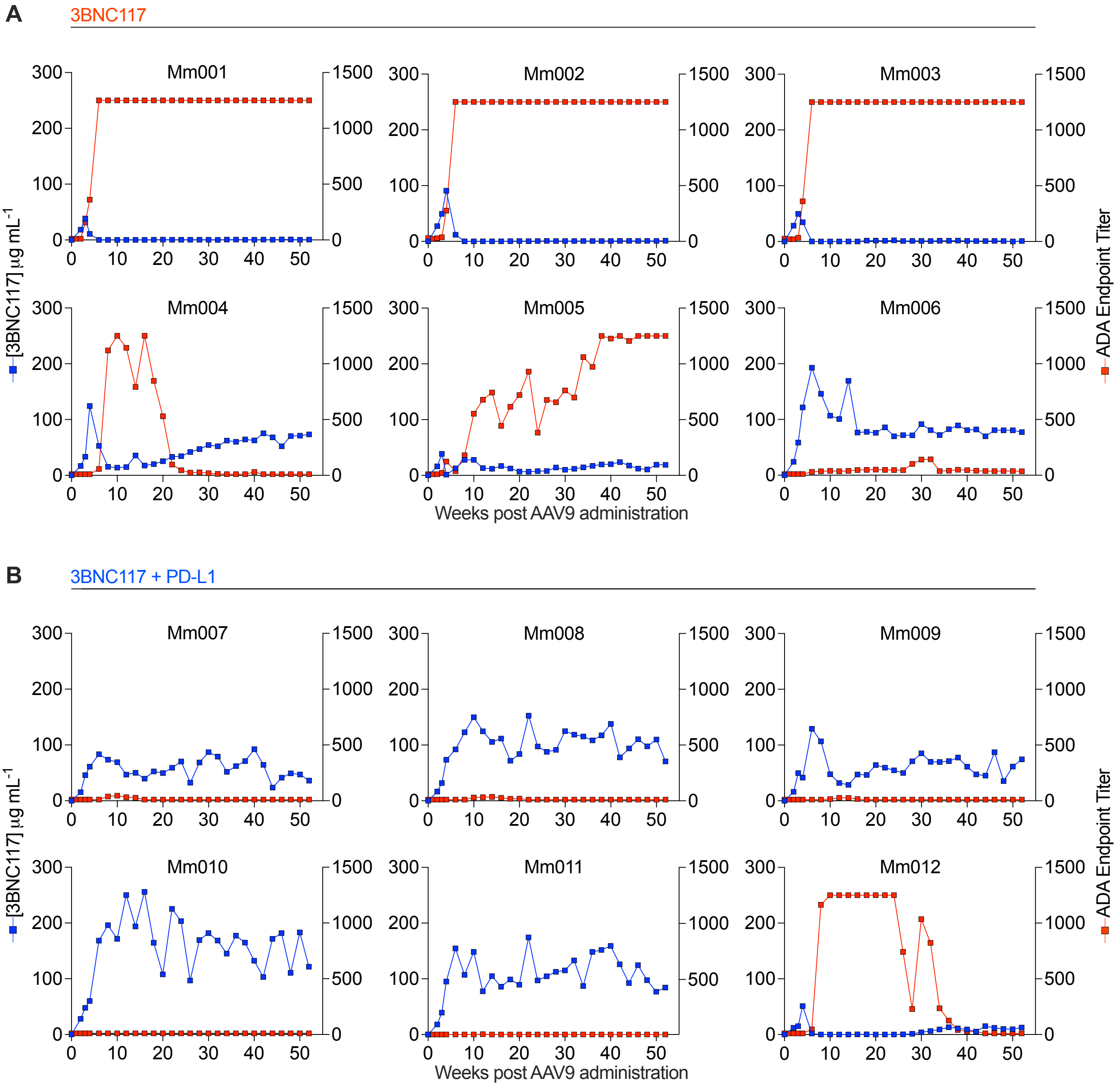


Kuipa et al., Fig. S3

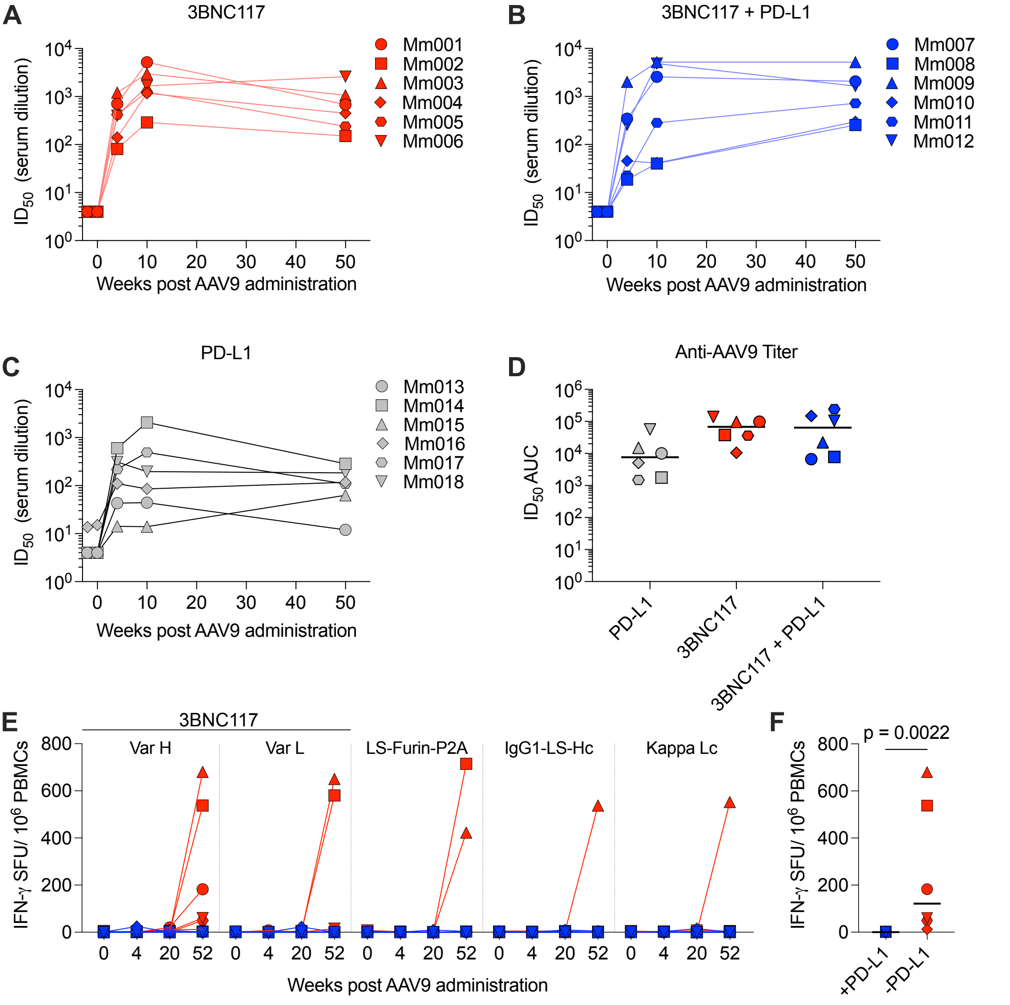


Kuipa et al., Fig. S4

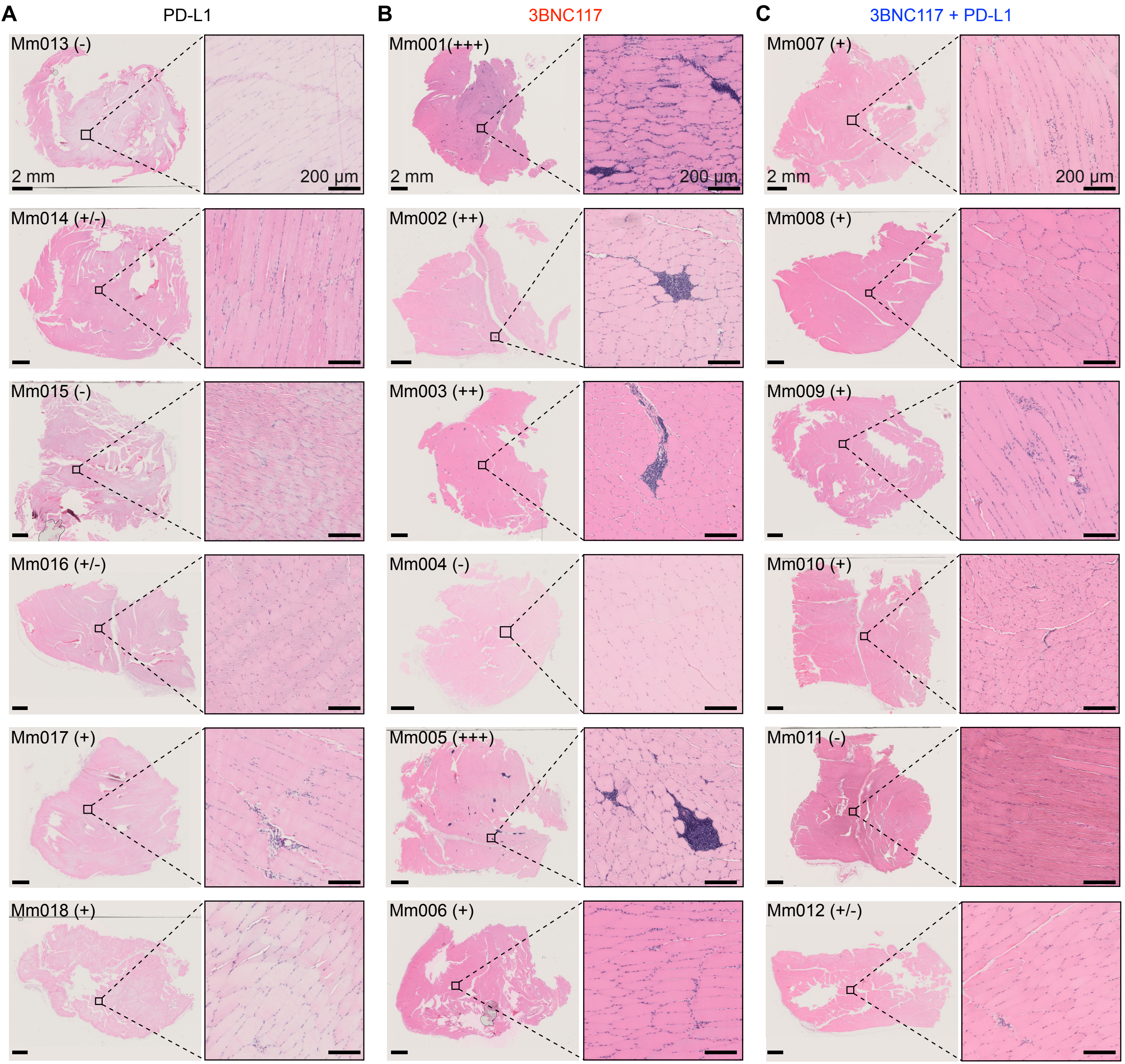


Kuipa et al., Fig. S5

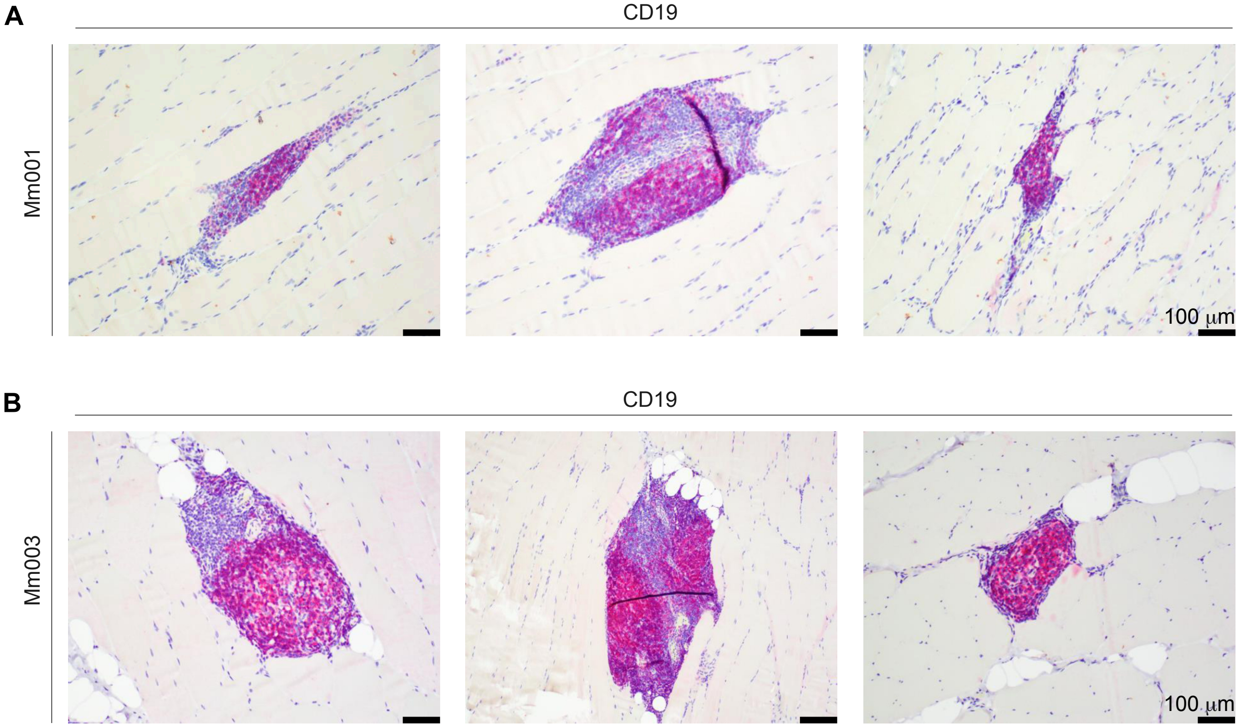


Kuipa et al., Fig. S6

| Macaque code | Group | Age (years) | Weight (kg) | Sex | *Mamu-B*17* | *Mamu-B*08* | *Mamu-A*01* | *Mamu-A*02* |
|---|---|---|---|---|---|---|---|---|
| Mm001 | 3BCN117-only | 3.2 | 5.3 | F | - | - | - | + |
| Mm002 | 3BCN117-only | 3.3 | 5.84 | M | - | - | - | - |
| Mm003 | 3BCN117-only | 3.6 | 6.18 | M | - | - | + | - |
| Mm004 | 3BCN117-only | 4.5 | 5.96 | F | - | - | - | + |
| Mm005 | 3BCN117-only | 3.5 | 6.17 | F | - | - | - | - |
| Mm006 | 3BCN117-only | 3.6 | 6.97 | M | - | - | - | - |
| Mm007 | 3BNC117 plus PD-L1 | 3.5 | 5.38 | F | - | - | - | - |
| Mm008 | 3BNC117 plus PD-L1 | 3.6 | 5.44 | F | - | - | - | - |
| Mm009 | 3BNC117 plus PD-L1 | 3.2 | 5.81 | M | - | - | - | - |
| Mm010 | 3BNC117 plus PD-L1 | 3.4 | 6.38 | M | - | - | - | - |
| Mm011 | 3BNC117 plus PD-L1 | 3.5 | 6.08 | F | - | - | - | - |
| Mm012 | 3BNC117 plus PD-L1 | 3.2 | 6.58 | M | - | - | - | - |
| Mm013 | PD-L1-only | 3.8 | 5.9 | M | - | - | - | + |
| Mm014 | PD-L1-only | 3.9 | 5.8 | F | - | - | + | - |
| Mm015 | PD-L1-only | 5.0 | 9.33 | M | - | - | - | - |
| Mm016 | PD-L1-only | 3.9 | 5.95 | M | - | - | - | - |
| Mm017 | PD-L1-only | 3.8 | 5.45 | M | - | - | - | - |
| Mm018 | PD-L1-only | 4.9 | 8.74 | M | - | - | - | - |

Kuipa et al., Table S1
